# Effects of Standing after a Meal on Glucose Metabolism and Energy Expenditure

**DOI:** 10.3390/ijerph20206934

**Published:** 2023-10-17

**Authors:** Hiroya Kono, Kento Furuta, Takumi Sakamoto, Shin-ya Ueda

**Affiliations:** 1 Graduate School of Education, Gifu University, Gifu 501-1193, Japan; 2 Graduate School of Health Science, Morinomiya University of Medical Sciences, Osaka 559-8611, Japan; 3 Faculty of Education, Gifu University, Gifu 501-1193, Japan

**Keywords:** sedentary time, standing desk, energy expenditure, postprandial glucose, exogenous glucose metabolic rate

## Abstract

In the past decade, university students have become more sedentary. A sedentary lifestyle is associated with an increased risk of obesity and cardiovascular disease. Methods that decrease sedentary lifestyles, such as the use of standing desks to increase physical activity, have been extensively examined. However, the effects of postprandial standing and sitting on energy metabolism have not yet been compared. Therefore, the present study investigated the effects of standing after a meal on energy expenditure and glucose metabolism. Ten males participated in the present study. The experiment was initiated with 300 g of rice ingested as a carbohydrate load. The subjects maintained a standing or sitting position for 120 min after the meal. Energy expenditure was calculated from VO_2_ and VCO_2_ using the indirect calorimetry method. Glucose metabolism was assessed by measuring blood glucose levels and the exogenous glucose metabolic rate. Energy expenditure through standing after eating was approximately 0.16 ± 0.08 kcal/min higher than that through sitting. Blood glucose dynamics did not significantly differ between the standing and sitting positions. Furthermore, no significant differences were observed in the dynamics of the exogenous glucose metabolic rate between the standing and sitting positions. Standing for 2 h after a meal increased energy expenditure by 10.7 ± 4.6% without affecting glucose metabolism.

## 1. Introduction

Obesity has been implicated in the development of several diseases, such as diabetes, cardiovascular disease, dyslipidemia, and hypertension [1]. Therefore, the prevention and attenuation of obesity may reduce the risk of these diseases. The fundamental cause of obesity is an energy imbalance between calories consumed and expended [1], and a sedentary lifestyle has been associated with obesity [2]. Therefore, sedentary behavior needs to be reduced and energy expenditure increased for the prevention of obesity.

However, university students unavoidably spend much of their time sitting. The time spent sitting by university students is longer than that by younger adults, and has been increasing in the past decade due to greater attendance at lectures, studying, and screen time [3]. In addition, lockdown measures due to the COVID-19 pandemic contributed to a more sedentary lifestyle in university students [4]. Therefore, the risk of obesity has recently increased among university students.

Prolonged sedentary behavior has been reported to increase the risk of developing type 2 diabetes, cardiovascular disease, and mortality [5]. The World Health Organization recommends engaging in moderate aerobic physical activity for 150 to 300 min per week and strength training at moderate intensity or higher at least twice a week to prevent these diseases [5]. However, sedentary times have been associated with obesity and cardiovascular disease independent of leisure-time physical activity [6,7].

Moreover, even in non-diabetic individuals, high postprandial blood glucose levels have been shown to promote oxidative stress, cause vascular endothelial dysfunction, and increase the risk of developing cardiovascular disease [8,9]. Low-grade physical activity equivalent to taking a short walk after meals, such as immediately attending to dishes and daily chores, was found to reduce postprandial blood glucose excursions [10]. Based on these findings, the prevention of diseases associated with sedentary behavior may be achieved by reducing sedentary times after meals and increasing physical activity.

Standing desks allow individuals to work while standing and have recently been promoted as a strategy to reduce sedentary behavior. The use of standing desks in school classrooms significantly decreased sedentary times by 18.3 min/day and significantly increased moderate-to-vigorous physical activity (MVPA) among primary school children [11]. The use of standing desks in the workplace also significantly increased MVPA [12]. Collectively, these findings demonstrate that the introduction of standing desks in school and work settings decreased sedentary times and increased MVPA.

Regarding the effects of using a standing desk on energy expenditure, 45 min of standing work after 12 h of fasting significantly increased energy expenditure by 0.34 ± 0.14 kcal/min from that in a sitting position [13]. Regarding the effects of using a standing desk on the postprandial glucose response, alternating bouts of sitting and standing by overweight and obese office workers significantly attenuated postprandial glucose responses [14]. Furthermore, university students reported that repeated standing and sitting every 20 min suppressed postprandial increases in blood glucose levels [15]. Therefore, maintaining a standing posture by utilizing a standing desk may decrease sitting times, increase energy metabolism from physical activity, and suppress postprandial elevations in blood glucose levels.

Prolonged standing has been recommended as a replacement to sitting to reduce cardiovascular risk [16,17]. However, standing for two hours was shown to increase pulse wave velocity [18], an indicator of arterial stiffness, while standing for more than 40 min per hour caused more pain and fatigue than sedentary work [19]. Although the duration of holding the standing position needs to be considered, the optimal duration of use of a standing desk with a focus on postprandial energy expenditure and glucose metabolism has not yet been examined.

A more detailed understanding of the effects of postprandial standing on energy expenditure and glucose metabolism over time will facilitate the control of the negative effects of prolonged standing, decrease sitting times among university students, and prevent diseases caused by a sedentary lifestyle. Therefore, the present study investigated the effects of postprandial standing on energy expenditure and glucose metabolism. We hypothesized that standing as an alternative to sitting after a meal reduces postprandial elevations in blood glucose and increases energy expenditure.

## 2. Materials and Methods

### 2.1. Participants

Fifteen young male subjects were recruited (age, 21.6 ± 1.1 years; height, 172.7 ± 5.8 cm; weight, 68.3 ± 5.9 kg). All subjects were lifelong non-smokers, and not on any medications, and none had any history of infectious disease for at least a 1-month period preceding this study. Subjects with a history of metabolic disorder or psychological diseases were excluded. This study was approved by the Ethics Committee of Morinomiya University of Medical Sciences (Admitting No. 2022-085 and 21 September 2022). All subjects provided written informed consent for participation in the present study.

### 2.2. Design

All subjects were required to complete two experimental trials (standing and sitting trials). Each trial was separated by 7 days and randomized. To control physical activity on the days before and on the mornings of the trials, subjects were instructed to refrain from moderate to intensive exercise for at least 24 h before each investigation. In addition, subjects were asked to refrain from consuming alcohol or caffeine from the day before until the end of the trial. Subjects were instructed to eat dinner by 9:00 p.m. on the day before the trial and then only drink water until breakfast. Subjects were also instructed to eat breakfast by 8:00 a.m. on the day of the trial. Each subject was instructed to eat the same breakfast in the two trials and then only drink water until testing. Subjects came to the laboratory at 12:00 p.m. and, after a 10 min rest period, consumed 300 g of rice as a carbohydrate (CHO) load. Subjects were then assigned to either sit at a traditional classroom desk or stand at a desk adjusted for height and hold the posture for 120 min.

The volumes of oxygen consumed (VO_2_) and carbon dioxide produced (VCO_2_), as well as RER, were measured using an automatic breath-by-breath respiratory gas analyzing system (AE-310S, Minato Medical Science, Osaka, Japan). Data were collected before rice consumption and for 10 min just before and 30, 60, 90, and 120 min after the start of the trial, with the first 7 min of each data collection being expunged to allow each subject to fully acclimate to the face mask worn for gas collection. Energy expenditure was calculated from VO_2_ and VCO_2_ using the indirect calorimetry method [20].

Before lunch and 30, 60, 90, and 120 min after the start of the trial, blood was collected with a puncture device from a fingertip to measure blood glucose levels using a self-monitoring glucometer (GLUCOCARD PlusCare, ARKRAY, Kyoto, Japan).

HR was continuously measured throughout experiments using an HR monitor (Polar V800, Polar Electro, Kempele, Finland).

To examine the use of energy substrates, we assessed CHO oxidation using a stable isotope of carbon. ^13^C-labeled glucose is mainly oxidized by working muscles during exercise and is subsequently excreted in expired gas as ^13^CO_2_. Therefore, the breath ^13^CO_2_/^12^CO_2_ ratio during exercise reflects the amount of exogenous glucose oxidized. Immediately after lunch, subjects were administered 100 mg ^13^C-glucose (D-Glucose-U-13C6 99%, Cambridge Isotope Laboratories, NJ, USA) dissolved in 100 mL of purified water. A baseline breath sample was collected using a 1.3-L sampling bag (Otsuka Pharmaceutical, Tokyo, Japan) before rice consumption. Breath samples were then collected after 30, 60, 90, and 120 min. The ^13^CO_2_/^12^CO_2_ ratio was determined to express the absolute increase between samples during exercise and the sample at baseline using an infrared spectrometer (POC One, Otsuka Pharmaceutical, Tokyo, Japan). The ^13^CO_2_ and ^12^CO_2_ abundance ratio was converted to the actual amount of excreted ^13^C, which was then applied to the formula below to evaluate ^13^C kinetics. ^13^C excretion per unit time was calculated as follows [21].

^13^C excretion = (Δ%^13^C/100) × 300 × BSA.

BSA is the body surface area.

BSA = (W0.425 × H0.725) × 0.007184.

where W is body weight measured in kilograms and H is body height measured in centimeters.

At the completion of the trial, RPE was assessed using the Borg scale [22].

### 2.3. Statistical Analysis

Statistical analyses were performed using SPSS for Windows (SPSS Inc., Chicago, IL, USA). All data were normally distributed and assessed by the Kolmogorov–Smirnov test. Data were presented as means ± S.D. Areas under the curve (AUC) were calculated using the trapezoidal rule to assess total changes in ^13^C excretion and blood glucose during each trial (t = 0 to t = 120). Hypothesis testing for the paired *t*-test was used to examine the significance of differences in average energy expenditure during the trial, and AUC and RPE at the end of the trial, between the standing and sitting positions. The other variables were analyzed using a two-way analysis of variance (ANOVA; repeated measures). When ANOVA revealed a significant interaction, the simple main effect was considered. When ANOVA did not reveal a significant interaction, the main effect was considered. If the simple main effect or main effect was revealed, a Bonferroni test was performed as a post hoc analysis to identify differences. *p* values < 0.05 were considered to be significant.

## 3. Results

### 3.1. Energy Expenditure and RER

**Figure 1:**
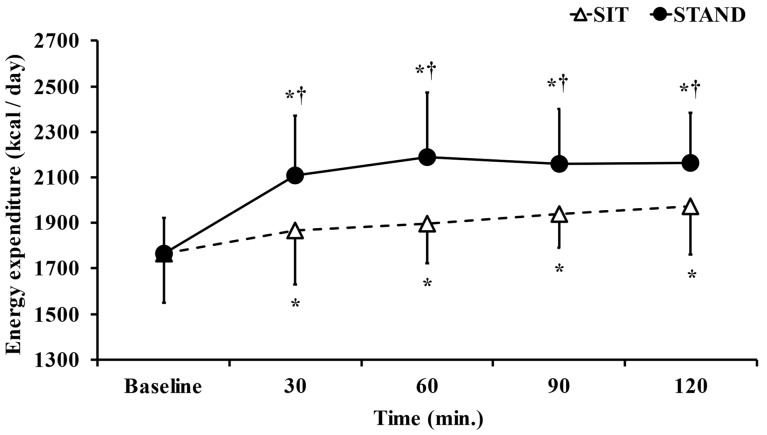
Comparison of energy expenditure dynamics in standing and sitting positions. Data are presented as the mean ± S.D. *n* = 15. * *p* < 0.05; vs. Baseline. ^†^ *p* < 0.05; vs. SIT.

The mean energy expenditure during the present study was significantly higher in the standing position than in the sitting position (*p* < 0.05, d = 1.15; Table 1). Figure 1 shows the dynamics of energy expenditure. In the two-way ANOVA, there were significant main effects of time (F = 32.68, *p* < 0.05, η^2^ = 0.714) and trial (F = 6.73, *p* < 0.05, η^2^ = 0.825) and a time× trial interaction (F = 6.58, *p* < 0.05, η^2^ = 0.307). In the post hoc test, energy expenditure was significantly higher in the standing position than in the sitting position 30, 60, 90, and 120 min after lunch (*p* < 0.05). Energy expenditure was significantly higher 30, 60, 90, and 120 min after lunch than at baseline in both the standing and sitting positions (*p* < 0.05).

**Figure 2:**
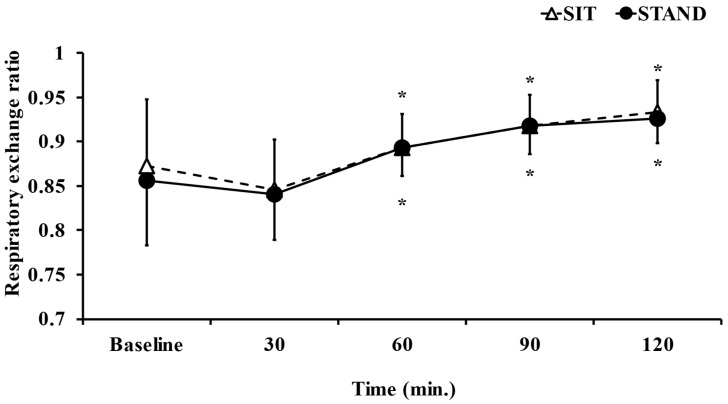
Comparison of dynamics of the respiratory exchange ratio in standing and sitting positions. Data are presented as the mean ± S.D. *n* = 15. * *p* < 0.05; vs. Baseline.

The mean RER during the present study did not significantly differ between the standing and sitting positions (*p* = 0.79, d = 0.089; Table 1). Figure 2 shows the dynamics of the RER. The two-way ANOVA revealed the significant main effect of time (F = 29.27, *p* < 0.05, η^2^ = 0.597). However, the main effect of trial (F = 0.16, *p* = 0.69, η^2^ = 0.018) and a time × trial interaction (F = 0.27, *p* = 0.89, η^2^ = 0.032) were not observed. In the post hoc test, RER was significantly higher 60, 90, and 120 min after lunch than at baseline in both the standing and sitting positions (*p* < 0.05).

### 3.2. Blood Glucose

**Figure 3:**
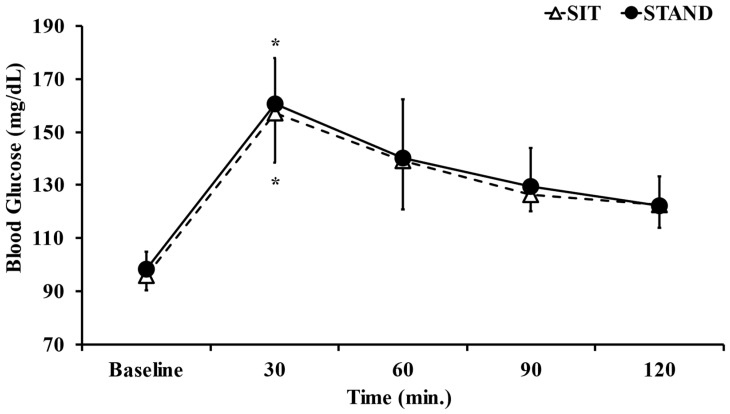
Comparison of blood glucose dynamics in standing and sitting positions. Data are presented as the mean ± S.D. *n* = 15. * *p* < 0.05; vs. Baseline, 60 min, 90 min, 120 min.

Figure 3 shows blood glucose dynamics. The two-way ANOVA showed the significant main effect of time (F = 92.08, *p* < 0.05, η^2^ = 0.819). However, the main effect of trial (F = 0.41, *p* = 0.52, η^2^ = 0.031) and a time × trial interaction (F = 0.11, *p* = 0.97, η^2^ = 0.016) were not observed. In the post hoc test, blood glucose levels were significantly higher 30 min after lunch than 60, 90, and 120 min after lunch in both the standing and sitting positions (*p* < 0.05). The AUC of blood glucose during the present study did not significantly differ between the standing and sitting positions (*p* = 0.49, d = 0.23; Table 1).

### 3.3. HR

**Figure 4:**
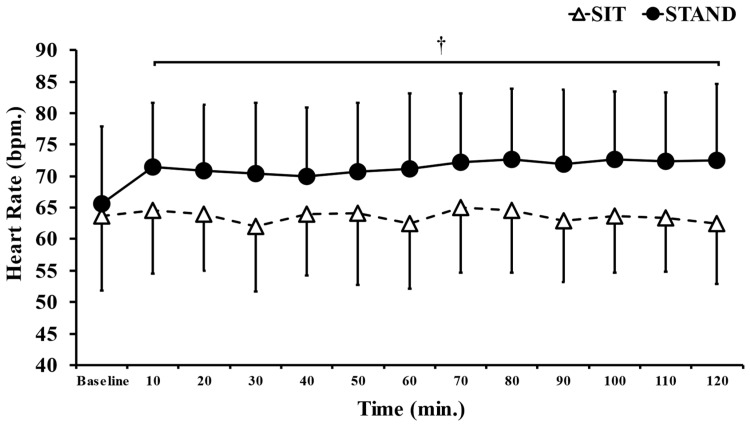
Comparison of heart rate dynamics in standing and sitting positions. Data are presented as the mean ± S.D. *n* = 15. ^†^ *p* < 0.05; vs. SIT.

The mean HR during the present study was significantly higher in the standing position than in the sitting position (*p* < 0.05, d = 0.77; Table 1). Figure 4 shows the dynamics of HR. The two-way ANOVA revealed the significant main effects of time (F = 5.31, *p* < 0.05, η^2^ = 0.238) and trial (F = 3.65, *p* < 0.05, η^2^ = 0.748) and a time × trial interaction (F = 31.27, *p* < 0.05, η^2^ = 0.304). In the post hoc test, HR was significantly higher in the standing position than in the sitting position from after the meal to the end of the experiment (*p* < 0.05).

### 3.4. Exogenous Glucose Metabolic Rate

**Figure 5:**
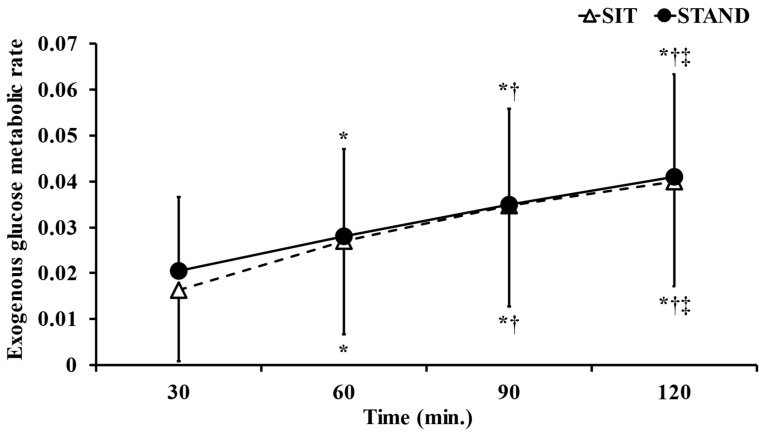
Comparison of dynamics of the exogenous glucose metabolic rate in standing and sitting positions. Data are presented as the mean ± S.D. *n* = 15. * *p* < 0.05; vs. 30 min. ^†^ *p* < 0.05; vs. 60 min. ^‡^ *p* < 0.05; vs. 90 min.

Figure 5 shows the dynamics of the exogenous glucose metabolic rate. The two-way ANOVA showed the significant main effect of time (F = 85.61, *p* < 0.05, η^2^ = 0.838). However, the main effect of trial (F = 0.053, *p* = 0.81, η^2^ = 0.011) and a time × trial interaction (F = 0.71, *p* = 0.54, η^2^ = 0.059) were not observed. In the post hoc test, the exogenous glucose metabolic rate incrementally and significantly increased over time (*p* < 0.05).

The AUC of the exogenous glucose metabolic rate during the present study did not significantly differ between the standing and sitting positions (*p* = 0.75, d = 0.069; Table 1).

### 3.5. RPE

RPE at the end of experiment was significantly higher in the standing position than in the sitting position (*p* < 0.05, d = 1.47; Table 1).

## 4. Discussion

The objective of the present study was to investigate the effects of standing after lunch on energy expenditure and glucose metabolism and compare them with those of sitting. The following results were obtained. (1) The mean energy expenditure after lunch was significantly higher in the standing position than in the sitting position by 10.7 ± 4.6%, and the dynamics of energy expenditure after lunch were significantly higher in the standing position than in the sitting position 30 min after lunch. (2) The exogenous glucose metabolic rate after lunch did not significantly differ between the standing and sitting positions. (3) The blood glucose response after lunch did not significantly differ between the standing and sitting positions.

The mean energy expenditure 120 min after lunch was 2154.2 ± 235.7 kcal/day in the standing position and 1918.1 ± 169.3 kcal/day in the sitting position, with that in the standing position being approximately 10.7 ± 4.6% higher than that in the sitting position. Physical activity ranged between 2.0 and 2.5 metabolic equivalents (METs) for standing and 1.5 METs for sitting, suggesting that standing was associated with higher energy expenditure than sitting due to increased physical activity [23]. Based on the present results, the substitution of 4 h of sitting per day with standing may result in an additional 38.4 kcal/day in energy expenditure, predicting a loss of 1.6 kg of body fat mass in one year. Moreover, the use of a standing desk has been shown to have no effect on subsequent energy consumption [24]. Therefore, replacing the sitting position with the standing position may prevent and attenuate obesity based on the principle of energy balance.

The dynamics of energy expenditure were significantly higher after lunch than at baseline in both the standing and sitting positions. A possible explanation for this increase is diet-induced thermogenesis (DIT), which is defined as an increase in the metabolic rate associated with the digestion, absorption, and storage of food and accounts for approximately 10–15% of total daily energy expenditure [25,26]. In the present study, the average energy expenditure was 9.3 ± 7.7% higher for sitting after lunch than at baseline, which is consistent with energy expenditure for DIT. Therefore, energy expenditure after lunch may have been increased by DIT in both the standing and sitting positions.

No significant differences were observed in energy expenditure between standing and sitting after lunch until 30 min after lunch. The mean difference in energy expenditure between standing and sitting in the present study was 0.16 ± 0.08 kcal/min. Therefore, to increase energy expenditure, the standing position needs to be maintained for approximately 30 min after eating.

In the present study, the dynamics of RER did not significantly differ between the standing and sitting positions, whereas RER was significantly higher 60, 90, and 120 min after lunch than at baseline in both the standing and sitting positions. In the present study, subjects consumed 300 g of rice as a CHO load. The body may preferentially metabolize increased CHO to maintain its internal environment. Prolonged low- to moderate-intensity exercise has been shown to increase lipid utilization [27]. However, 120 min of standing after a meal did not decrease RER in the present study.

Exercise increases the uptake of glucose into contracting skeletal muscle by translocating glucose transporter type 4 independently of insulin [28]. Although Dobashi et al. (2021) reported that alternating work postures suppressed postprandial blood glucose elevations more than sitting work postures, the present results showed no significant differences in blood glucose dynamics between the standing and sitting positions [15]. Regarding muscle contractions, alternating work postures involves muscle contractions with knee flexion and extension in the switching movement between the standing and sitting positions; however, the present study did not include this movement between the standing and sitting positions. Harris et al. (1981) reported that glucose metabolism also increased in isometric contractions without knee flexion but required isometric contractions of 66% of a maximum voluntary contraction of the quadriceps muscle [29]. These findings suggest that the standing position in the present study did not increase glucose uptake due to insufficient muscle contraction [30]. It is impractical for the body to apply isometric contractions of 66% of a maximum voluntary contraction during a lecture, study, or other task. Furthermore, postprandial blood glucose levels peak 30 min after a meal. Therefore, dynamic contractions of skeletal muscles, such as exercise involving knee flexion, need to be performed before 30 min after a meal in order to control postprandial increases in blood glucose levels.

Karpovich demonstrated that the HRs in adults was approximately 7 bpm higher in the standing position than in the sitting position [31], which is consistent with the present results showing HRs of 71.6 ± 11 bpm in the standing position and 63.6 ± 9.6 bpm in the sitting position in the 120 min after lunch.

No significant differences were observed in the exogenous glucose metabolic rate between the standing and sitting positions. In normal subjects, the dynamics of postprandial plasma glucose concentrations were shown to peak 30–60 min after a meal and return to pre-prandial values 2–3 h after a meal [32]. Insulin secretion was elevated by increasing glucose levels, which promoted glucose uptake from blood into muscles and reduced plasma glucose levels [32]. The increase observed in the exogenous glucose metabolic rate after lunch in the present study is consistent with these findings. The present study also suggested that glucose ingested immediately before standing work was metabolized in the muscles for at least 2 h. Furthermore, no significant difference in glucose metabolism was observed between the standing and sitting positions, indicating that the difference in energy expenditure between the standing and sitting positions was due to the preferential use of lipids. Since subjective exercise intensity was significantly higher at the end of the standing position than in the sitting position, increased physical activity appeared to have affected subjective exercise intensity.

There are some limitations that need to be addressed. We recruited a small number of subjects in the present study. A large population study will be necessary to confirm the present study. In particular, subjects in the present study were young, healthy males; therefore, it remains unclear whether the results obtained may be generalized to other populations, such as females, the elderly, or overweight and obese individuals. Subjects were not unified in how they spent their time in the standing and sitting positions. In the same sitting posture, a difference of 0.5 METs was noted between no activity and doing some work. Therefore, it is necessary to unify work in future studies. Moreover, since the experiment was initiated immediately after the meal had been consumed, we were unable to examine the timing at which the standing position was the most effective. Further studies to measure energy metabolism in the standing position before eating and in the standing position after a certain amount of rest after eating are needed in order to investigate the effects of the timing of standing on energy expenditure. Knee flexion exercises after eating may contribute to the suppression of postprandial hyperglycemia. In the future, a more detailed understanding of the timing of the switch between the standing and sitting positions may assist in the development of new exercise programs to prevent and treat diseases caused by an increased sedentary lifestyle, such as obesity, arteriosclerosis, and cardiovascular disease.

## 5. Conclusions

In conclusion, the present study showed that energy expenditure after lunch was approximately 10% higher in the standing position than in the sitting position and also that the predominant energy substrate was lipids. Furthermore, contractions of skeletal muscles, such as those involving knee flexion, appeared to be required to increase glucose metabolism. Since significant differences were observed in energy expenditure when the standing position alone was held until 30 min after a meal, the RPE was higher in the standing position, and postprandial blood glucose levels peaked 30 min after a meal. Therefore, the best approach to utilizing a standing desk after a meal is to repeatedly switch between standing and seated work positions within 30 min after a meal. The present results may serve as a basis for promoting shorter postprandial sitting times.

## Figures and Tables

**Table 1:** Mean value of each measured item during the study.

	Sit	Stand	*p* Value
Energy expenditure (kcal/day)	1918.1 ± 169.3	2154.2 ± 235.7	*p* < 0.05
RER ^a^	0.9 ± 0.03	0.89 ± 0.03	*p* = 0.79
AUC ^b^ of blood glucose (min mg/dL)	15,947 ± 889	16,221 ± 1416.4	*p* = 0.49
HR ^c^ (bpm.)	63.6 ± 9.6	71.6 ± 11	*p* < 0.05
AUC ^b^ of exogenous glucose metabolic rate	2.7 ± 1.8	2.8 ± 1.7	*p* = 0.75
RPE ^d^	8.1 ± 1.8	11.3 ± 2.4	*p* < 0.05

Data are presented as the mean ± S.D. *p* values < 0.05 were considered to be significant. *n* = 15. ^a^ Respiratory exchange ratio. ^b^ Area under the curve. ^c^ Heart rate ^d^ Rating of Perceived Exertion.

## Data Availability

The data presented in this study are available on request from the corresponding author.
